# Human inflammatory bowel disease does not associate with *Lawsonia intracellularis *infection

**DOI:** 10.1186/1471-2180-6-81

**Published:** 2006-09-19

**Authors:** Christoph W Michalski, Fabio Francesco Di Mola, Klaus Kümmel, Michael Wendt, Jörg S Köninger, Thomas Giese, Nathalia A Giese, Helmut Friess

**Affiliations:** 1 Department of General Surgery, University of Heidelberg, Im Neuenheimer Feld 110, D-69210 Heidelberg, Germany; 2 Clinic for Pigs, Small Ruminants, Forensic Medicine and Ambulatory Service, University of Veterinary Medicine, Bünteweg 2, D-30559 Hannover, Germany; 3 Institute of Immunology, University of Heidelberg, Im Neuenheimer Feld 305, D-69120 Heidelberg, Germany

## Abstract

**Background:**

There is increasing evidence that bacterial infection of the intestinal mucosa may contribute to the pathogenesis of inflammatory bowel diseases (IBD). In pigs, an obligate intracellular bacterium, Lawsonia intracellularis (LI), was shown to cause proliferative enteropathy (PE) of which some forms display histological and clinical similarities to human IBD. Since LI-similar *Desulfovibrio spp*. may infect human cells, we hypothesized that LI might be associated with the development of human IBD.

**Results:**

In human intestinal tissue samples, PCR using LLG, 50SL27, LSA and strictly LI-specific 16SII primers, yielded either no amplicons or products with weak homology to human genomic sequences. Sequencing of these amplicons revealed no specificity for LI. However, amplification of DNA with less specific 16SI primers resulted in products bearing homology to certain Streptococcus species. These 16SI-amplified products were present in healthy and diseased specimens, without obvious prevalence.

**Conclusion:**

LI is not associated with the pathogenesis of UC or CD. Whether an immunologic response to commensal bacteria such as streptococci may contribute to the chronic inflammatory condition in IBD, remained to be determined.

## Background

Inflammatory bowel diseases (IBD) comprises primarily Crohn's disease (CD) and ulcerative colitis (UC) which are characterized by recurrent inflammatory attacks associated with tissue destruction [1,2]. The etiology of these diseases is multifactorial and genetic or infectious factors have been postulated to be part of the formation of the chronically inflammatory condition [3-5]. Besides some contributing genetic factors such as NOD2/CARD15 variants in CD patients, there seems to be an impairment in the intestinal mucosal barrier function leading to defective immunoregulation [6-8]. Furthermore, there is increasing evidence that bacterial infection of the intestinal mucosa may be involved in its pathogenesis [5]. In particular, Mycobacterium paratuberculosis has been found in tissue and blood samples of CD patients, though the question whether mucosal dysregulation leads to infection or vice versa remains unanswered [4,9,10]. It is also unclear whether this bacterium is an etiologic factor or is detected solely by coincidence, and thus has no influence on the course of the disease. In UC, there is evidence for a cross-talk between commensal bacteria and the mucosal immune system while the exact role of these bacteria also has to be further elucidated [5].

The obligate intracellular bacterium Lawsonia intracellularis (LI) is the causative agent of porcine proliferative enteropathy (PE) [11,12]which mainly affects young animals and occurs in either a chronic inflammatory course (porcine intestinal adenomatosis, PIA; necrotising enteritis; regional ileitis) or in an acute form (proliferative hemorrhagic enteropathy) [13-16]. However, PIA is the main form of PE, for which proliferation but not inflammation is the major histological finding. Inflammation occurs in the more necrotic, complicated forms, such as the above mentioned necrotising enteritis and regional ileitis. In veterinary medicine, PE has gained importance mainly because of economic reasons due to death loss, increased medication costs and decreased food conversion of the infected animals [14]. It was shown that LI may be the causative agent for PE in other species such as hamster, deer and ostrich [11,17] indicating that it is a pathogen with a wide host range. In several occupational correlation studies, a potential link between Lawsonia intracellularis and Crohn's disease was not found [18,19]. Immunohistochemistries of colonic biopsies from ulcerative colitis patients were also negative for LI [20]. Sera of IBD patients were negative for the presence of agglutinins to the common secondary agent of PE, Campylobacter mucosalis [21]. Furthermore, the slight clinical similarities of PE and human IBD were not confirmed in histological comparison analyses which showed marked differences between these disease entities [22-26]. Population studies found agricultural production of livestock to rather have a protective effect for Crohn's disease [19]. These differences in enteric pathology and the lack of epidemiological evidence lead many authors to the assumption that the association was probably negative. However, a PCR-based search for molecular evidence of L. intracellularis or related species in human tissue specimens has not been conducted so far. Due to slight clinical similarities, we still hypothesized that LI may be involved in the pathogenesis of human IBD. Conventional and quantitative PCR using specific primer pairs were employed to detect LI DNA in operative specimens of CD, UC and normal intestinal tissue samples.

## Results

Intestinal DNA preparations were subjected to PCR after isolation from CD, UC, normal tissue samples and pig faeces (positive control). Five primer sets were used for consequent PCR detection of LI DNA, assuming that different genes may be conserved differently across LI strains infecting various species or in the *Desulfovibrio *group [17,27]. First, we attempted to detect LI DNA in human ileum samples by PCR using previously published primer sets with proven specificity (Table 2).

**Figure 1:**
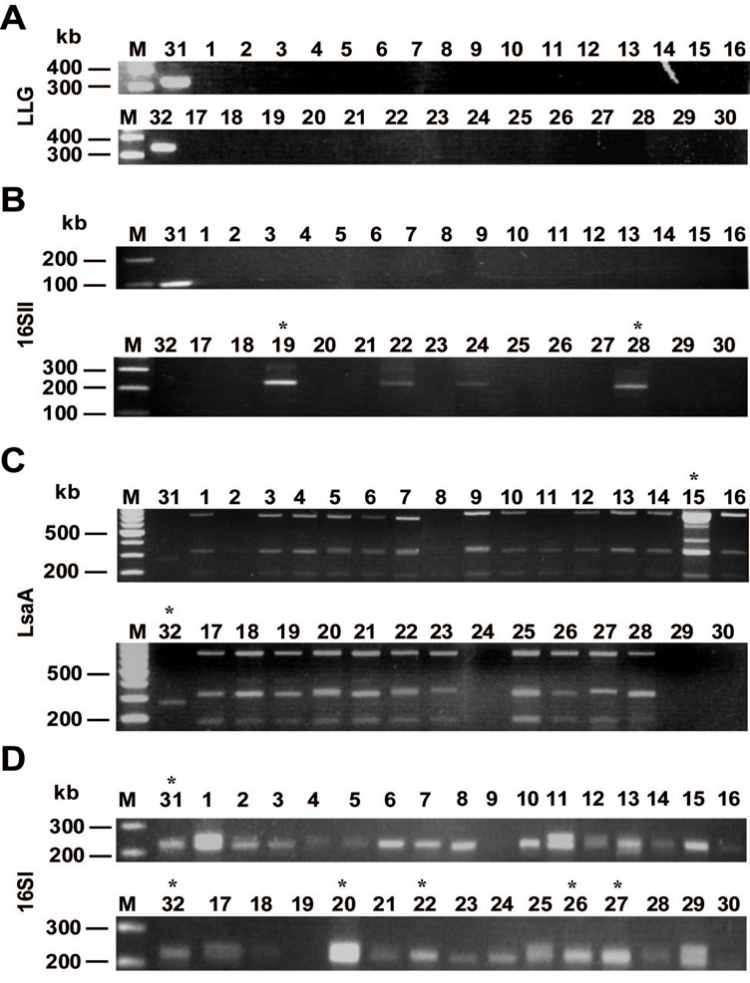
**Detection of *Lawsonia intracellularis *DNA in intestinal specimens by PCR using different sets of primers**. Bacterial DNA was prepared from intestinal specimens obtained from organ donors and IBD patients as described in Material and Methods, and PCR-amplified using primers recognizing lyase-like gene (LLG, A), 16S ribosomal subunit (set II, B and set I, D), and Lawsonia surface antigen (LsaA, C). The sample numeration is as described in Table 1. (31, 32) depict porcine fecal DNA samples used as positive controls; (*) – samples used for sequencing.

The most widely tested LI-specific primers designed by Jones et al. [28] re located within a lyase-like gene of LI (LLG), according to our BLAST search. This set did not yield a signal in any of the analyzed tissue samples (Fig. 1A). Only DNA preparations from pig faeces produced a correctly-sized amplicon.

The use of another pair of published primers recognizing 16S ribosomal subunit (16SII; [29]) produced a correctly-sized product confirmed by sequencing LI-specific amplicon (98 bp) only for the positive control. The fragment of 210 bp size seen in two CD and two diverticulitis samples (#19, 22, 24 and 28) was apparently a result of non-specific amplification of host gDNA, according to the sequencing analysis.

The newly designed primers recognizing another gene – LsaA – amplified one single band in the porcine positive controls with the size of 280 base pairs, although calculation based on the deposited sequence AF498259 yielded 153 bp (Fig. 1C). Sequencing analysis showed 124 bp-long LI-homology stretch. Since the sequencing of the full LI genome is not completed yet, we can just speculate that either the deposited sequence is lacking a fragment, or we detected a LI subtype in the particular pigs. Moreover, LsaA primers uniformly produced three fragments of different sizes, approximately 750 bp, 330 bp and 180 base pairs in all tested samples. None of these 3 fragments, separately excised and purified from electrophoretically separated sample #15, had similarity to the published LI or any other bacterial sequence.

The primers recognizing 50SL27 did not produce a signal in any of intestinal samples, but a correctly sized one in swine faeces (data not shown), resembling LLG-specific PCR profile.

**Figure 2:**
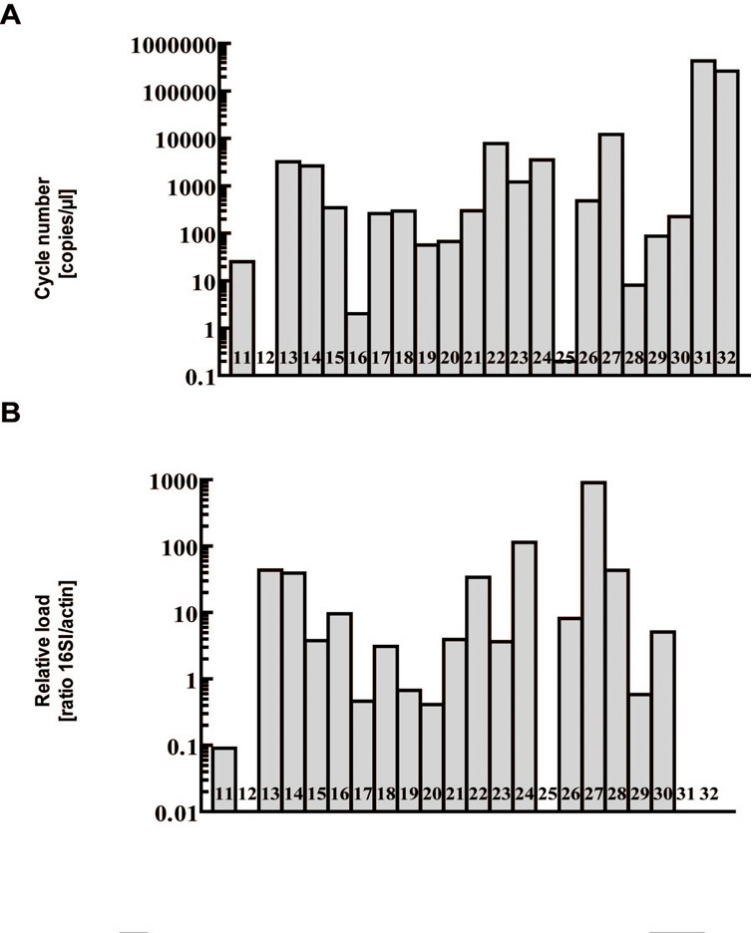
**Quantification of 16SI-amplified products by Q-PCR analysis of intestinal DNA preparation**. Bacterial DNA was prepared from intestinal specimens obtained from IBD patients as described in Material and Methods, pre-amplified for 10 cycles, diluted 1:100 and further amplified in LightCycler using primers recognizing bacterial 16S subunit (16SI). Data are presented as an absolute number of copies per μl of input DNA (A) or as relative bacterial load calculated as ratio of 16SI-amplicons to β-actin amplicons (B). Note that this calculation is not applicable in the case of porcine fecal samples.

In contrast, primers designed for a conserved region of 16S (16SI), produced correctly sized 237 bp-long positive control as well as a fragment of about the same size in all other DNA preparations, including normal tissues. This fragment was slightly varying in size between intestinal samples (± 10 bp), and was occasionally accompanied by a second band of approximately 260 bp. Interestingly, heterogeneous band appearance was observed among different samples obtained from the same patient (#11–14). 16SI primers allowed detecting an amplicon with the expected size of 237 bp in almost all analyzed samples (Fig. 1D). The PCR products representing two CD (#15, 26/27) and one diverticulitis (#22) cases were sequenced and analyzed using BLAST, the megaBLAST and the discontigous megaBLAST-homology search. The alignments revealed more than 90% homology of the amplicons obtained from one diverticulitis and two CD samples (##22, 26 and 15, respectively) and approximately 18% homology of CD sample #27 with different Streptococcus species such as S. sanguinis, S. parasanguinis, S. oralis, S. mitis, S. gordonii, S. intermedius, S. gallolyticus, S. milleri, S. iniae, S. anginosus and several uncultured streptococcaceae strains but no similarity with the 16S ribosomal subunit of LI. Since 16SI-amplified product was seen in almost all reactions, we sought to determine whether bacterial load may be associated with disease outbreak. To this end, quantitative PCR was performed with the 16SI primers. Once the samples amplified by Q-PCR were subsequently analyzed by gel-electrophoresis (data not shown), they demonstrated the same pattern as shown after conventional PCR in Fig. 1D. Used as a negative control, a commercially available genomic DNA preparation (Roche) did dot produce any signal. However, only six samples gave a signal valid for quantifying. Therefore, DNA preparations were first subjected to 10 cycles of PCR pre-amplification, then diluted 1:100 and used for a new round of PCR in LightCycler. This approach allowed quantification in all samples, yet revealed no definite disease-specific pattern among analyzed samples (Fig. 2).

## Discussion and conclusion

Bacterial infection of the intestinal mucosa may play an important role in the etiology of human ulcerative colitis (UC) and Crohn's disease (CD). However, it is unclear whether infection precedes disordered immunoregulation or vice versa.

In 1913, Dalziel reported that in Scotland animal paratuberculosis, known as Johne's disease, and human chronic granulomatosis, now known as Crohn's disease, exhibit histopathological and clinical similarities [30]. Therefore, a number of studies examined the role of M. paratuberculosis, the causative agent of Johne's disease, in CD [2,4,31,32] and revealed positive evidence for the involvement of M. paratuberculosis in CD [4,5,9]. In UC, there is evidence for an abnormal immunological response to commensal bacteria of the intestinal microflora, but no specific bacterium has been identified so far as the causative agent [2,3,33]. Since porcine proliferative enteropathy (PE) was showing certain similarities to human IBD, and, *Desulfovibrio *was found to seed human tissues (GenBank accession No. U42221; [34]), we sought to determine whether LI may also be a causative agent in human CD or UC.

PCR was carried out with primers designed for amplification of different LI genes with various degree of conservation: the 16S ribosomal subunit (designated 16SI and 16SII), the 50S ribosomal subunit L27 (50SL27), the lyase-like gene (LLG) and the lawsonia specific surface antigen A (LsaA). The commercial use of some of these previously published primers with high sensitivity and specificity suggests that the PCR method may detect a possible LI infection in human tissues [17,28,29,35,36]. However, there was no LI-specific DNA amplification in human intestinal samples. Unfortunately, control samples derived from humans exposed to pigs were not available for analysis. However, since no association was found between PE and human IBD we abstained from including such control samples in our analysis. An alignment of several amplicons obtained with the 16SI primers (detecting besides LI 16S ribosomal subunit also 16S ribosomal subunits in *Desulfovibrio *spp. and other bacteria) from inflamed bowel samples revealed the presence of highly similar 16S ribosomal sequences of streptococcus species. There is evidence that Crohn's disease is associated with an aberrant inflammatory response to bacterial antigens [8,37,38] caused by a persistent antigenic stimulation in genetically susceptible patients. Also, colonization of macrophages in the lamina propria may induce persistance of inflammation and thus chronic enteritis [38]. Furthermore, presence of 16S rDNA (highly conserved among various bacteria species) was shown in normal intestinal tissues and in inflamed intestinal biopsies of CD patients [39,40]. Both virulent and non-virulent streptococci are implicated in the pathogenesis of periodontal/gingival inflammation [41]. Since chronic intestinal inflammation does not develop in bacteria-free mice, an involvement of streptococci in the pathogenesis of intestinal inflammation may be possible either as part of the commensal flora (non-pathogenic) or as an infectious and pathogenic agent. The small size of the patient's population may have compounded limitation of Q-PCR analysis, which did not show particular bacterial prevalence in IBD samples used in the present study.

Though LI seems not to be involved in the pathogenesis of Crohn's disease or ulcerative colitis, it may be worth to further investigate a possible involvement of porcine or cattle bacteria in their etiology. Intensive mass animal farming, increasing meat consumption and a rising incidence of CD and UC in industrialized countries point towards common mechanisms of action of animal and human IBD. Furthermore, M. paratuberculosis may not be the only animal pathogen involved in the development and progression of these diseases.

In conclusion, despite some similarities between PE and IBD, the swine causative agent – *Lawsonia intracellularis*, does not appear to associate with human disease. However, an involvement of different streptococcus species in the IBD pathogenesis is worthy of further exploration.

## Methods

### Patients and tissue sampling

30 intestinal tissue samples were obtained intraoperatively from 10 organ donors and 13 patients comprising 9 patients with IBD, two with diverticulitis, one with sigmoid volvulus and one with pancreatic cancer. Multiple sampling from different intestinal locations was performed in some patients. Table 1 summarizes the data of the patients whose tissues were analyzed. The use of human tissues was approved by the Ethical Committee University of Bern (Switzerland), and written informed consent was obtained from each patient. In all cases, the diagnosis was confirmed according to the conventional clinical and histopathological criteria [42] at the Department of Pathology University of Bern (Switzerland). Two porcine faecal samples were obtained from pigs with clinically and histopathologically proven porcine proliferative enteropathy. Tissue samples were immediately immersed in liquid nitrogen and stored at -80°C until subsequent DNA preparation and analysis.

### Isolation of bacterial DNA

All reagents and equipment for bacterial DNA preparation were purchased from Roche Applied Science (Mannheim, Germany). Human tissue and porcine stool samples were incubated in Bacteria Lysis Buffer (part of MagnaPure LC DNA preparation kitIII for bacteria and fungi) supplemented with proteinaseK for 10 min at 65°C and subsequently at 56°C over night. Following five freeze-boil cycles (using N_2 _and a heating block), the samples were further disintegrated using the Ribolyzer instrument, then supplemented with 0.01% dithiotreitol and transferred to the MagnaPure LC sample cartridge for automatic DNA isolation.

### Primer design and Polymerase Chain Reaction

To detect LI in specimens, DNA was subjected to conventional and real-time PCR (Q-PCR) analyses. For conventional PCR, bacterial DNA was amplified using Taq-polymerase (Invitrogen) in a gradient cycler (MJ Research) by 35 cycles of heating at 94°C for 1 min, annealing at 54°C for 45s and elongation at 72°C for 1 min. All reagents and equipment for Q-PCR were purchased from Roche Applied Science (Mannheim, Germany). Q-PCR was performed with the LightCycler Fast Start DNA SYBR Green kit according to established protocols [10]. In order to compare the level of LI between samples, the amount of specific transcripts was related to the level of sample's genomic DNA and expressed as LI to β-actin ratio.

PCR amplification was performed using 5 pairs of LI-specific primers recognizing different genes of the bacterium and included previously published and verified as well as newly designed sets (using the Oligo 6.1 software package; Table 2). The genes of interest are frequently used as targets for primers in the diagnosis of porcine PE and the primer design was based on the published sequences of these genes as well as the entire Lawsonis genome which is available on the website of the National Center for Biotechnology Information (NCBI; ) and the Kyoto Encyclopedia of Genes and Genomes (KEGG) [12,17,27-29,35,43,44]. As to 16S ribosomal subunit, 2 sets were employed: a strictly LI-specific 16SII [29] and less specific 16SI. Following PCR, ethidium bromide staining was used to visualize PCR products after agarose gel electrophoresis of the samples.

### Sequencing and sequence analysis

The identity of PCR products was established via sequencing performed by Qiagen Sequencing Services (Hilden, Germany) using PCR-matching primers. If more than one fragment was seen upon ethidium bromide staining of electrophoretically separated samples, each differently sized band was excised from the gel and purified (gel-purification kit for PCR products, Qiagen) prior to the sequencing. Obtained sequences were subjected to the homology search analysis using the NCBI databases and BLAST algorithm [45].

## Abbreviations

IBD: inflammatory bowel diseases; UC: ulcerative colitis; CD: Crohn's disease; LI: Lawsonia intracellularis; PE: porcine proliferative enteropathy; PIA: porcine intestinal adenomatosis; BLAST: ; LsaA: Lawsonia specific surface antigen A; LLG: lyase-like gene

## Authors' contributions

CWM carried out the preparation of samples, molecular analyses, participated in the sequence alignments and drafted the manuscript. FFDM carried out the conventional PCR analyses and participated in drafting the manuscript. KK and MW participated in the preparation of samples, in the primer design and in drafting the manuscript. JSK and TG carried out the quantitative RT-PCR analyses, designed the corresponding primers and evaluated the resulting data. NG and HF participated in designing the study, performed the statistical analyses and coordinated and helped to draft the manuscript. All authors read and approved the final manuscript.

## Figures and Tables

**Table 1:** Patient information for tissue samples

***sample no***.	***diagnosis***	***inflammation***	***clinical comments***
1–10, 29	Organ donor	none	none
19–20, 22	Diverticulitis	acute	Colon resection
25	Sigmoid volvulus	acute	Colon resection
11–21, 23, 24, 26–28, 30	IBD	Chronic; mean disease duration: 8.9 yrs	Operation due to highly active disease and/or abdominal pain

31	porcine PE, faeces		positive control
32	porcine PE, faeces		

**Table 2:** LI-specific primer sets

**primer set**	**primer sequence**	**target accession no**.	**expected size (bp)**	**design**
16SI-5'	AGTGGCGCACGGGTGAGTAAC	U30147	237	this paper, [44], [46]
16SI-3'	TCCAGTGTGGCCGATTATCCT			
16SII-5'	GCGCGCGTAGGTGGTTATAT	L15739	98	Lindecrona 2002, [29]
16SII-3'	GCCACCCTCTCCGATACTCA			
LLG	TATGGCTGTCAAACACTCCG	L08049	329	Jones 1993, [28]
LLG	TGAAGGTATTGGTATTCTCC			
LsaA-5'	GTTTACGCTTTAGATGTTGGTAACAAT	AF498259	153	this paper, [27]
LsaA-3'	AATAAATGAAACATCGCAAACTACTT			
50S L27-5'	CTAGTTGACGAACAAGGATATTGCC	U45241	114	this paper
50S L27-3'	AGAAAGCTGGTGGAAGTTCTCGC			
